# Challenges in High-grade Neuroendocrine Neoplasms and Mixed Neuroendocrine/Non-neuroendocrine Neoplasms

**DOI:** 10.1007/s12022-021-09676-z

**Published:** 2021-03-31

**Authors:** Stefano La Rosa

**Affiliations:** Institute of Pathology, University Hospital and University of Lausanne, Lausanne, Switzerland

**Keywords:** Neuroendocrine tumor, High grade, Ki67, Neuroendocrine carcinoma, MiNEN, Diagnosis

## Abstract

The growth in knowledge of the pathogenesis, molecular background, and immunohistochemical profile of neuroendocrine neoplasms (NENs) has led not only to an increased awareness of these diseases but also to several changes of the nomenclature. In particular, the concept and terminology of high-grade (grade 3) NENs and mixed neoplasms have changed considerably over the last 20 years, creating some confusion among pathologists and clinicians. The aim of this review is to elucidate the diagnostic criteria, including the most important differential diagnoses of high-grade NENs and mixed neuroendocrine/non-neuroendocrine neoplasms (MiNENs). The role of the Ki67 labelling index and morphology, used to define grade 3 NENs of the digestive system and lungs, is also discussed. The evolution of the concepts and terminology of MiNENs is revised, including the most important differential diagnoses.

## Introduction

The last 20 years have seen considerable growth in the knowledge of neuroendocrine neoplasms (NENs) leading not only to an increased awareness of these diseases but also to several changes in the nomenclature, mainly due to a better understanding of their pathogenesis, molecular background, and clinical behavior. Although this evolution has clearly improved patient management, it has created some confusion among pathologists and clinicians, especially if not working in referral centers dedicated to neuroendocrine pathology. The concepts and terminology of high-grade NENs and mixed neoplasms have changed considerably. Hence, the aim of this review is to elucidate their diagnostic criteria, molecular-morphologic correlates, and prognostic stratification.

## High-grade NENs

### Evolution of Their Understanding

**Fig. 1:**
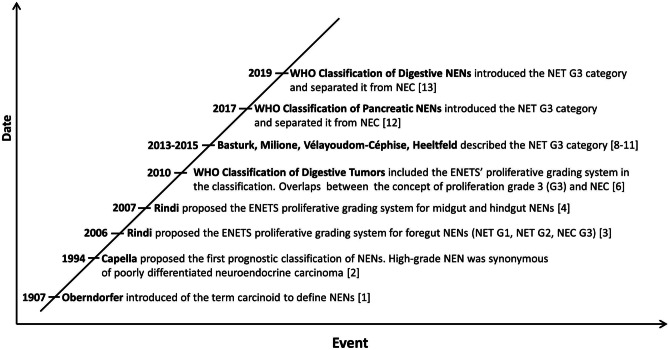
Timeline of the evolution of the concept of high-grade neuroendocrine neoplasms of the digestive system. ENETS, European Neuroendocrine Tumor Society; NEN, neuroendocrine neoplasm; NET, neuroendocrine tumor; NEC, neuroendocrine carcinoma

Although NENs have been well known since 1907, when Oberndorfer introduced the term “carcinoid” [1], the concept of high-grade NENs is more recent and has been better developed during the last 15 years (Fig. 1). In 1994, the first prognostic classification of NENs was proposed, and poorly differentiated neuroendocrine carcinomas were clearly separated from well differentiated neuroendocrine tumors to underline their different biological aggressiveness and prognosis [2]. Since then, the term high-grade NEN has been synonymous with poorly differentiated neuroendocrine carcinoma. It was only in 2006, 100 years after the introduction of the term carcinoid, that the concept of grade for digestive NENs was proposed by the European Neuroendocrine Tumor Society (ENETS). It was based on cell proliferation and evaluated using mitotic count and the Ki67 proliferative index [3, 4]. Based on the growing body of evidence concerning the prognostic importance of proliferation [5], the WHO classification of digestive NENs, published in 2010, adopted the ENETS’ three-tiered grading system, with the first two grades (G1 and G2) being included in the well differentiated tumor categories and G3 representing poorly differentiated neuroendocrine carcinomas (NEC) [6]. A weak point of this approach was the complete apparent overlap between morphological differentiation and proliferation grading, which was revealed to be a critical point. Indeed, the existence of morphologically well differentiated NENs, called neuroendocrine tumors (NETs), with a high proliferation index (mitotic count > 20 mitoses/2 mm^2^ and/or Ki67 index > 20%, grade 3) was not included in the 2010 WHO classification [6]. However, starting from clinical observations [7], it soon became evident that NENs with a high proliferation index (high-grade NENs) are morphologically, clinically, and biologically heterogeneous [8–11]. Consequently, the concept of NET G3 (well differentiated tumors with high proliferation) was successively integrated into the WHO classifications of GEP neoplasms, leaving the definition of NEC to NENs with poorly differentiated morphology [12, 13]. Similar observations have been described in thoracic sites [14], where a proliferative grading system has still not been officially adopted in the WHO classification [15]. The fact that NETs (including NET G3) and NECs are distinct clinico-pathological entities has been supported for a long time [16], and molecular findings have confirmed this assumption, showing that these two families of neoplasms follow different pathogenetic pathways [17]. However, very recent evidence suggests that a subgroup of large cell neuroendocrine carcinomas probably arise from pre-existing NETs in both respiratory and digestive systems [14, 18, 19], although this phenomenon needs to be better explored.

### High-grade NENs of the Digestive System

#### Definition and Diagnostic Criteria

By definition, high-grade NENs include neoplasms of proliferative grade 3, characterized by a mitotic count > 20 mitoses/2 mm^2^ and/or a Ki67 proliferative index > 20%. Morphologically, they can be either well differentiated (NET G3) or poorly differentiated (NEC) (Table 1). This distinction is fundamental due to their different prognoses (median overall survival of 33 months for NETs G3 versus 11 months for NECs) [9, 20], clinical presentation, functional imaging characterization, and different therapeutic approaches [21, 22]. Indeed, NETs G3 can generally be identified by somatostatin receptor imaging, whereas in NECs, which show loss of morphological differentiation and increase of proliferation, ^18^F-FDG represents the best imaging procedure [21]. NETs G3 are generally treated like the more common NETs G2 using different approaches, which include surgery (for localized disease) and different medical options such as chemotherapy, everolimus, and peptide receptor radionucleotide therapy (PRRT). Conversely, surgery has a limited role for NECs, while the treatment of choice is platinum-based chemotherapy [21, 22].

**Table 1:** WHO classification of digestive neuroendocrine neoplasms [12, 13]

	Morphological differentiation	Proliferative grade	Mitotic count/2 mm^2^	Ki67 index
NET G1	Well-differentiated	Grade 1	< 2	< 3%
NET G2	Well-differentiated	Grade 2	2–20	3–20%
NET G3	Well-differentiated	Grade 3	> 20	> 20%
NEC	Poorly differentiated	Grade 3	> 20	> 20%
MiNENs	Well or poorly differentiated	Variable	Variable	Variable

*NET* neuroendocrine tumor, *NEC* neuroendocrine carcinoma, *MiNEN* mixed neuroendocrine/non-neuroendocrine neoplasm

NETs G3 are histologically characterized by an organoid proliferation of rather uniform cells, with moderately abundant granular and eosinophilic cytoplasm. Nuclei are generally round, with finely granular (“salt and pepper”) chromatin and small nucleoli, more prominent in some cases. Nuclear atypia may be up to moderate in some cases. Vascular and lymphatic invasion is frequently observed as well as perineural invasion. By definition, these NETs show high proliferation including > 20 mitoses/2 mm^2^ or, more frequently, a Ki67 proliferative index > 20%. The Ki67 index seems more reliable for defining NETs G3, which although higher than 20%, generally do not exceed 55–60% as observed in NECs. Tumor cells are positive for general neuroendocrine markers including synaptophysin, chromogranin A, and INSM1. They also express somatostatin receptor 2A and can be positive for CDX2, ISL1, PDX1, and different hormones (i.e., insulin, glucagon, somatostatin, pancreatic polypeptide, gastrin, serotonin, substance P, glicentin, and PYY) depending on the site of origin [16]. The role of immunohistochemistry for transcription factors and hormones is useful for the correct characterization of these neoplasms and also for the identification of their primary site when they present as metastatic disease without a known primary origin [23]. Indeed, transcription factor and hormone expression generally reflects the site of origin as summarized in Table 2. It is worth noting that the expression of transcription factors in NECs does not reflect the origin and, consequently, cannot be used to identify the site of origin of metastatic occult NECs [23].

**Table 2:** Correlation between the site of origin and transcription factor and hormone expression in lung and digestive well-differentiated neuroendocrine tumors (NETs)

Site of origin of NET	Transcription factors	Site-specific hormones	Other markers (hormones or others)
Lung	TTF1 (+/−) OTP	GRP/bombesin Serotonin	Calcitonin α-hCG
Stomach	CDX2 (−/ +)	Histamine* Ghrelin Serotonin Gastrin Somatostatin (rare)	VMAT2
Duodenum	PDX1 ISL1 CDX2 (+/−)	Somatostatin Gastrin Serotonin	Insulin (very rare)
Ileum and appendix	CDX2	Serotonin	VMAT1 VMAT2 Substance P S100 (sustentacular cells)
Rectum	ISL1 SATB2	Glicentin Pancreatic polypeptide PYY Somatostatin Serotonin	PSAP
Pancreas	PDX1 CDX2 (+/−) ISL1	Insulin Glucagon Pancreatic polypeptide Somatostatin	Gastrin Neurotensin Calcitonin Serotonin

*TTF1* thyroid transcription factor-1, *OTP* orthopedia homeobox protein, *CDX2* caudal type homeobox 2; *PDX1* pancreatic and duodenal homeobox 1, *ISL1* insulin gene enhancer binding protein Isl-1, *VMAT* vesicular monoamine transporter, *PSAP* prostatic acid phosphatase

*Not commercially available antibodies well working

**Fig. 2:**
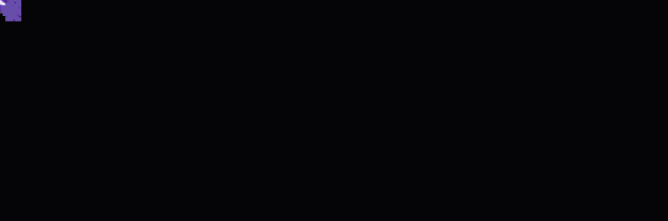
The distinction between small cell **A** and large cell **B** neuroendocrine carcinoma is based on morphology. Small cell neuroendocrine carcinoma is composed of round to oval, small- to medium-sized cells, measuring 2–4 times the size of a small lymphocyte. The cytoplasm is scant and the nuclei are hyperchromatic with inconspicuous nucleoli. Conversely, large cell neuroendocrine carcinomas are composed of large cells with vesicular nuclei showing prominent nucleoli and abundant eosinophilic cytoplasm

NECs are generally larger neoplasms showing deep infiltration of the bowel wall or of the peri-pancreatic tissue (in the pancreas). Microscopically, they are characterized by a solid proliferation of cells with large areas of “geographic chart” necrosis. They can be divided into the small and large cell subtypes, based on the morphological features of neoplastic cells. Small cell carcinomas (Fig. 2a) are composed of small to medium-sized (2–4 times the size of a small lymphocyte), round to oval cells with scant cytoplasm, indistinct cell borders, and hyperchromatic nuclei with inconspicuous nucleoli. Large cell subtypes (Fig. 2b) are composed of large cells with vesicular nuclei showing prominent nucleoli and abundant eosinophilic cytoplasm, which form a more structured organoid architecture than small cell NECs. In both large cell and small cell subtypes, mitotic figures are extremely frequent, as well as apoptotic bodies and vascular and perineural infiltration. The neuroendocrine nature needs to be confirmed by immunohistochemical analyses and includes the expression of general neuroendocrine markers: synaptophysin and INSM1 are generally well expressed, while chromogranin A can be negative or focally positive, typically showing a perinuclear dot-like pattern of immunoreactivity. Tumor cells can express TTF1 and CDX2 irrespective of the site of origin and can be positive for ASH1 [16, 24].

Interestingly, the distribution of NETs G3 and NECs in the digestive system is different. NETs G3 have been more frequently observed in the pancreas [20]. They have also been described in the stomach, more commonly as a subgroup of type 3 NETs, although type 1 ECL-cell G3 NETs have also been rarely reported [25, 26]. They are rare in other digestive sites including the midgut and rectum [20]. Conversely, NECs seem to be more frequent than NETs G3 in the gut than in the pancreas and, in particular, in the rectum followed by the stomach [20, 25].

#### Mimickers and Differential Diagnosis

The correct diagnosis of a NET G3 and its differential diagnosis with G1/G2 NETs, NECs, or other non-neuroendocrine mimickers are fundamental for the specific therapeutic approaches of these different entities.

**Fig. 3:**
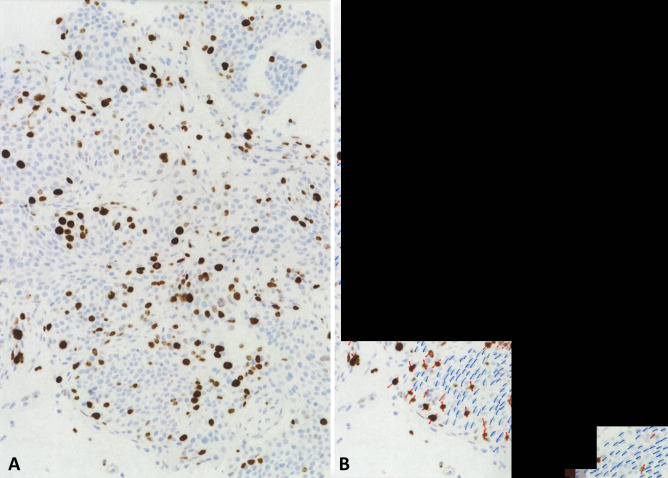
Evaluation of Ki67 proliferative index by manual count on a camera-captured, printed image **A**. This field, selected at low power magnification, represents the highest labeled area (“hot spot”) of the tumor. On this image, all the Ki67-positive (marked in red) and Ki67-negative (marked in bleu) tumor nuclei were counted, excluding endothelial, stromal and lymphocytic cells **B**. 268 nuclei were Ki67 positive on 1146 counted tumor nuclei, corresponding to a Ki67 proliferative index of 23%

Due to the morphological overlap among NETs, irrespective of their grade, the diagnosis of NET G3 respect to NET G1 and G2 is based on the accurate evaluation of the Ki67 proliferative index. Different methods for the evaluation of the Ki67 labelling index have been proposed and used in previous years. However, the manual count on a camera-captured printed image appears to be the most reliable procedure [27, 28] and for this reason was officially proposed by the WHO [12]. In practice, the highest labeled area (“hot spot”) is selected, and a picture is taken and printed (Fig. 3a). All the Ki67-positive neoplastic nuclei need to be counted from this image, regardless of the staining intensity or whether the nuclei show a speckled or a diffuse stain (Fig. 3b). The number of stained nuclei is then expressed as a percentage (“index”) of immunoreactive cells in 500 to 1000 tumor cells. This method has been demonstrated to show good reproducibility and takes on average between 10 and 15 min. Other methods have been proposed but do not seem to perform as well, as discussed elsewhere [5].

The differential diagnosis between NET G3 and small cell NEC is generally easy and based on the morphological characteristics of tumor cells and does not require specific and sophisticated additional techniques.

**Fig. 4:**
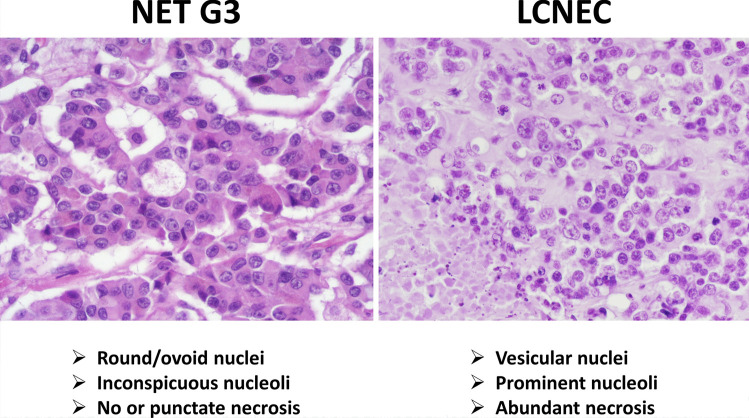
Morphological features useful for differentiating between grade 3 neuroendocrine tumors (NET G3) and large cell neuroendocrine carcinomas (LCNEC)

**Fig. 5:**
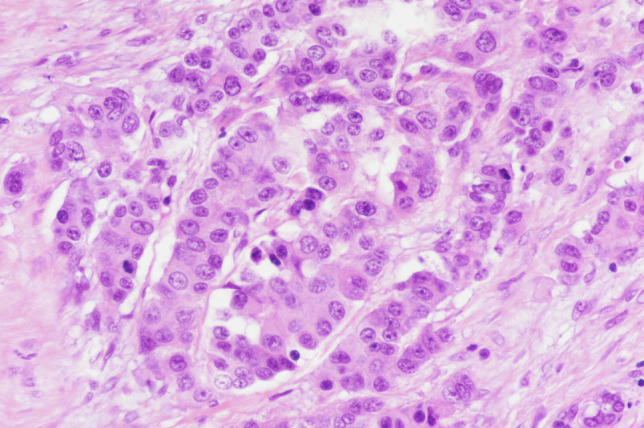
This NET G3 shows some clear nuclei with evident nucleoli in the absence of necrosis and true vesicular and large nuclei with prominent eosinophilic nucleoli. This “borderline cell morphology” renders the differential diagnosis with a large cell neuroendocrine carcinoma difficult. In similar cases, the use of additional immunohistochemical and/or molecular analyses is useful (see the text)

The differential diagnosis between NET G3 and large cell NEC (LCNEC) is first based on morphological grounds, once the neuroendocrine nature is confirmed by the appropriate immunohistochemical profile. It is generally easy due to the different morphological characteristics of tumor cells (Fig. 4). However, in some cases, the morphology of NET G3 may be less clear showing “borderline” features including some nuclei with vesicular chromatin and evident nucleolus or showing pleomorphic features (Fig. 5). In these cases, the use of additional immunohistochemical markers and/or molecular analysis is recommended. It is well known that the molecular background of NETs is different from that of NECs and this directly reflects the different expression of several markers [17, 29]. Indeed, the carcinogenesis of NECs is similar to that of non-neuroendocrine carcinomas of the primary site in which they arise and includes inactivation of *TP53* and *RB1* [17]. In contrast, NETs of the digestive system can present inactivation of *MEN1*, *VHL*, and *TSC1/2* genes and the hyperactivation of the PI3K/mTOR pathway [29]. For these reasons, NETs G3 generally show a “wild-type” p53 expression and diffuse immunoreactivity for Rb protein in neoplastic nuclei, reflecting the absence of mutation in the relatives genes. ATRX and DAXX expression can be lost in about 30% of pancreatic NETs as a consequence of their gene mutations as well as MEN1 protein expression. In addition to these markers, whose immunohistochemical expression strongly correlates with gene mutations, the expression of chromogranin A and somatostatin receptor 2A can be different between NETs G3 and LCNECs [23]. Chromogranin A is strongly and diffusely positive in NETs, while it can be focal with a perinuclear “dot-like” pattern of immunoreactivity in NECs. Somatostatin receptor 2A shows strong membrane expression in NETs (score 3+ according to Volante et al.) [30], while in NECs, its expression can be focal and cytoplasmic, or even absent.

**Fig. 6:**
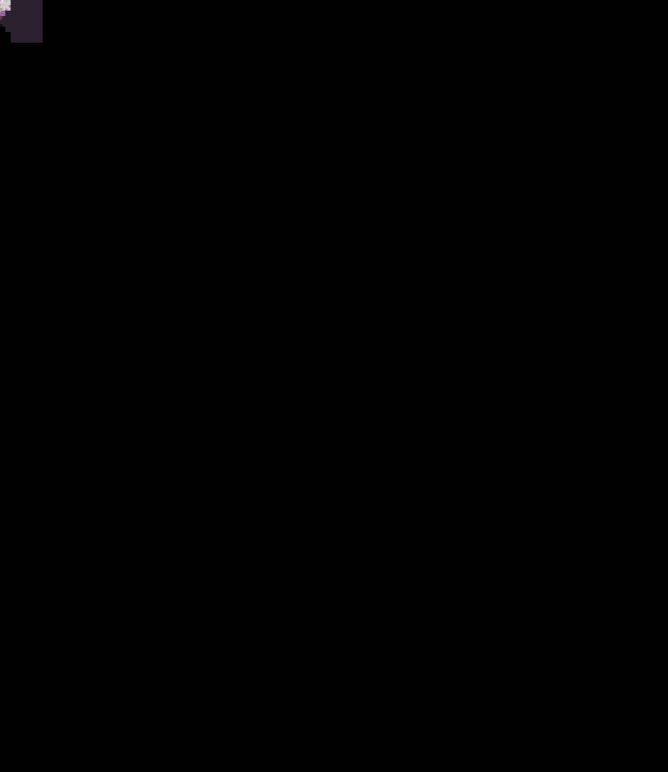
Pancreatic acinar cell carcinomas may simulate a NET G3. This is an example of a pancreatic neoplasm with neuroendocrine-like features including a trabecular pattern of growth **A**. However, in some areas, abundant necrosis is observed **B** and at high power magnification tumor cells show nuclei with prominent nucleoli **C** and high mitotic count **D**. In addition, a high Ki67 index is found **E** and neuroendocrine markers are lacking (**F**, synaptophysin). All these features should suggest the possible differential diagnosis with an acinar cell carcinoma, and an immunohistochemical analysis including BCL10 and trypsin is mandatory. This case was positive for both acinar cell markers, and the final diagnosis was acinar cell carcinoma

Among non-neuroendocrine mimickers, acinar cell carcinoma (ACC) represents an important potential pitfall when diagnosing a pancreatic NET G3. The morphological suspicion of an ACC should be taken into account when a tumor showing a well-differentiated neuroendocrine-like morphology presents areas of abundant necrosis, unexpected prominent nucleoli, very high mitotic count or Ki67 index, and focal or absent expression of general neuroendocrine markers (Fig. 6). In these cases, an additional immunohistochemical panel including BCL10 and trypsin is mandatory to confirm the diagnosis of ACC.

The differential diagnosis with an adenocarcinoma showing a solid/organoid growth pattern should be considered when a neoplasm with a neuroendocrine-like morphology and composed of medium- to large-sized cells is completely negative for general neuroendocrine markers.

### High-grade NENs of the Lung

#### Definition and Diagnostic Criteria

**Fig. 7:**
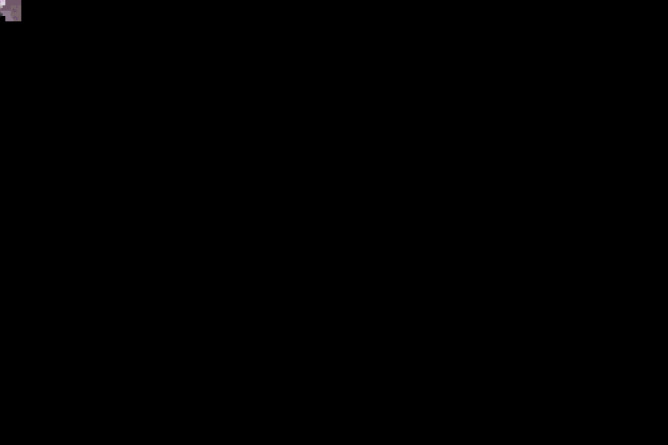
NET G3 of the lung. This tumor, defined as atypical carcinoid following the WHO criteria [7], shows a trabecular architecture and is composed of well differentiated cells. Two mitoses (arrows) are present in this field **A**. Tumor cells are positive for synaptophysin **B** and the Ki67 proliferative index is higher than 25% **C**. Immunohistochemistry for p53 shows scattered and weak positive nuclei

The recognition and definition of high-grade carcinoids (NETs) in the lung are not as well established as in the digestive system. Indeed, for years, the classification of lung NETs (carcinoids), also including their distinction from LCNECs, has been based on morphological criteria, which included mitotic count and the presence of necrosis. The Ki67 proliferative index was only introduced in the last WHO classification of lung NENs as an ancillary marker useful in the diagnostic work-up of lung or bronchial biopsies [15]. However, the Ki67 proliferative index has progressively emerged as a relevant prognostic indicator in lung carcinoids [31] and, consequently, its assessment has been strongly recommended [32]. In line with these observations, Rindi and coworkers proposed a prognostic grading system, which, in addition to mitotic index and necrosis, included Ki67 index [33]. However, this grading system has not been universally accepted [34], although its clinical value also appeared useful in the pre-operative setting [35]. Irrespective of the integration of Ki67 into a grading system, an increasing body of evidence has demonstrated that a high Ki67 index (using a cutoff of 10% or 25%) identifies a subgroup of lung carcinoids (NETs) with a worse outcome, which is unrelated to their typical or atypical subtype. It has been suggested that these cases may represent the lung counterpart of the well-known digestive “NET G3,” since they retain a well differentiated morphology associated with high mitotic and Ki67 index (Fig. 7) in the absence of *RB1* and *TP53* alterations [36–38]. With respect to the digestive system, the Ki67 cutoffs of 10% and 25% seem better at separating NET G1 from NET G2 and NET G2 from NET G3, respectively [39]. Taken together, these findings support the hypothesis that, similarly to the digestive system, thoracic NENs can include NETs with different grades (from G1 to G3) and NECs of large and small cell subtypes that fall into the poorly differentiated category and show a Ki67 index > 55% (Table 3) [39].

**Table 3:** Proposal of a prognostic classification of lung neuroendocrine neoplasms [39]

Morphological differentiation	Ki67 proliferative index	Proposed terminology	WHO terminology [7]
Well differentiated	Ki67 ≤ 10%	NET G1	Typical carcinoid—frequent Atypical carcinoid—rare
Well differentiated	10% < Ki67 ≤ 25%	NET G2	Typical carcinoid—rare Atypical carcinoid—frequent
Well differentiated	25% < Ki67 ≤ 55%	NET G3	Atypical carcinoid
Poorly differentiated	Ki67 > 55%	NEC	Large or small cell NECs

#### Mimickers and Differential Diagnosis

Although still not considered as a separate entity as in the digestive system, lung carcinoids (NETs) with high proliferation, which can be provisionally considered “lung NETs G3,” need to be recognized for their worse prognosis than not highly proliferative carcinoids and better prognosis than LCNECs. The distinction from “low proliferative” carcinoid types is based on the count of Ki67-positive nuclei. Although standardized criteria to evaluate the Ki67 index have not been validated for lung NETs, it appears reasonable that the same system for the evaluation of the Ki67 proliferative index validated in the digestive system can be used (see the specific paragraph above). Consequently, despite similar morphological features, lung G3 NETs can easily be identified and separated from G1 and G2 NETs using Ki67 immunohistochemistry. The distinction of G3 NETs from LCNECs may be more problematic in some cases and the use of molecular tests can be of help, namely the search for *RB1* or *TP53* mutations. It is worth noting that LCNECs generally show a Ki67 proliferative index higher than 50–60%, which is rarely found in carcinoids.

## MiNENs

### Evolution of Their Understanding and Definition

Neoplasms showing the coexistence of a neuroendocrine and a non-neuroendocrine component have been described in almost all organs of the human body and have been a matter of investigation during the last 20 years. In particular, their clinical relevance has increased since the use of immunohistochemistry has been largely introduced in the work-up of tumors, facilitating the possibility of their identification with a consequent increase in diagnosis.

Their terminology as well as their inclusion in the WHO classifications of tumors of different sites have been a matter of discussion for years. Indeed, different terms have been used for defining mixed neoplasms composed of both neuroendocrine and non-neuroendocrine components creating some confusion. Although described in all systems, they have been better characterized and investigated in the digestive system, where they were called “mixed endocrine-exocrine tumors” (MEEC) in the WHO classification published in 2000 [40]. In 2010, the term MEEC was replaced by the term “mixed adenoneuroendocrine carcinoma” (MANEC) [6], which was criticized in subsequent years. The most important weak point of this terminology resides in the fact that it states that all neoplasms are composed of adenocarcinomas and NECs; although this represents the most frequent association, it does not encompass the large spectrum of possible combinations. Indeed, both neuroendocrine and non-neuroendocrine components can show variable morphological features: NECs or NETs can represent the neuroendocrine component, while the histological features of the non-neuroendocrine component depend on the site of origin (adenocarcinoma, squamous cell carcinoma, acinar cell carcinoma, etc.). In addition, the term MANEC cannot be applied to mixed neoplasms arising in other systems, including endocrine and non-endocrine organs like the pituitary gland, lung, skin, and those of the urogenital system and head and neck region. To cover this wide spectrum of different mixed neoplasms, also showing some site-related peculiarities, in 2016, we proposed the simple and general term “mixed neuroendocrine/non-neuroendocrine neoplasm (MiNEN)” [41]. This term has the advantage of being used for diagnosing mixed neoplasms arising in different organs and resulting from the combination of different components. Consequently, it can be used as a diagnostic term in pathology reports, with the addition of a detailed description of the two neoplastic components to provide the clinician with the prognostic information useful for choosing the most appropriate therapy. The term MiNEN has been accepted and is in use for the diagnosis of mixed neoplasms arising in the digestive system [12, 13]. However, it has not been officially accepted for mixed neoplasms arising in other sites including prostate, urinary bladder, and kidney [42]. In addition, such mixed neoplasms are not classified as separated entities in several organs, where they are included as subtypes of neuroendocrine carcinomas due to the similar clinical behavior (i.e., in the lung and larynx) [15, 43] or are not classified at all due to their rarity (for example in the nasal cavity, pituitary gland or skin) [43–45]. We suggest that the term MiNEN could also be used to define mixed neoplasms arising in non-digestive organs, in line with the recent proposition of a common classification framework to unify the terminology of NENs arising in different organs [46].

MiNENs are neoplasms in which the two components are clonally related. Consequently, independent neuroendocrine and non-neuroendocrine neoplasms arising in the same organ and abutting one another are excluded. For these cases, the term “collision tumor” is recommended. Several molecular studies have recently demonstrated that MiNENs show genetically related components, irrespective of their morphology [47–55].

### MiNENs of the Digestive System

#### Diagnostic Criteria and Classification

**Fig. 8:**
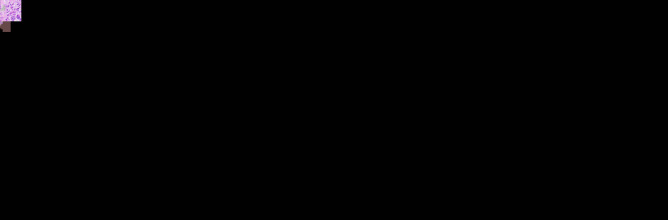
Mixed neuroendocrine/non-neuroendocrine neoplasm (MiNEN) is a neoplasm in which the two components represent at least 30% of the tumor burden **A**. Adenocarcinoma with interspersed chromogranin-positive neuroendocrine cells **B** is not considered MiNEN

Following the WHO classification [13], digestive MiNENs are neoplasms in which the two components are malignant, are morphologically and immunohistochemically recognizable, and each of them represents at least 30% of the tumor burden (Fig. 8a). However, it is worth noting that the 30% cutoff has been chosen arbitrarily and was not based on proven clinical evidence [56]. This cutoff was first chosen to underline that a minor neuroendocrine component in an adenocarcinoma does not influence the prognosis. However, in light of recent knowledge and improved morphological definitions, it may be critically reconsidered. The fact that adenocarcinomas or squamous cell carcinomas with scattered neuroendocrine cells (Fig. 8b), only identifiable with immunohistochemistry, cannot be included into the MiNEN category does not represent a matter for discussion, since, by definition, different tumor components need to be morphologically recognizable and the presence of scattered neuroendocrine cells does not have any clinical meaning. Conversely, the discussion is open for neoplasms in which the two components are recognizable and have a clonal origin, even when not reaching 30%. They may be considered MiNENs, and this is also justified from a clinical point of view, especially when one of the two components is represented by an aggressive cancer (i.e., NEC), which influences the prognosis irrespective of its amount. Consequently, the maintenance of the 30% cutoff does not now appear mandatory for defining MiNENs. A modern classification approach, which takes into account the recent molecular knowledge of these neoplasms, may eliminate this rule in all cases in which the different tumor components are recognizable and clonally related [57], as established in other organs (i.e., lung) where no minimum percentage of either component is required to define a neoplasm as mixed [15].

#### Mimickers and Differential Diagnosis

The distinction of a MiNENs from an adenocarcinoma with interspersed neuroendocrine cells is rather simple because the neuroendocrine component is not morphologically recognizable and neuroendocrine cells are only identified using immunohistochemistry.

However, the differential diagnosis of an amphicrine carcinoma, which may morphologically resemble a MiNEN, may be more problematic. Amphicrine carcinomas, which can be found in both digestive and extra-digestive sites, are peculiar “hybrid neoplasms” showing morphological, immunohistochemical, and ultrastructural features of both neuroendocrine and exocrine differentiation in the same cells [57]. The hallmark of the differential diagnosis resides in the lack of typical organoid and neuroendocrine-like morphology associated with the co-expression of neuroendocrine and non-neuroendocrine markers in the same cells. From a clinical point of view, amphicrine carcinomas resemble exocrine carcinomas of the same site and recent molecular findings demonstrated that they share similarities with adenocarcinomas, but not with NENs [58].

Another important differential diagnosis includes the so-called goblet cell carcinoids of the appendix. A large amount of clinical and molecular information has demonstrated that these peculiar neoplasms are adenocarcinomas with interspersed neuroendocrine cells and for this reason they are now considered a peculiar variant of appendiceal adenocarcinoma defined as “goblet cell adenocarcinoma” [59].

The WHO definition of MiNEN does not include neoplasms in which the non-neuroendocrine component is not malignant, such as an adenoma, due to the negligible clinical impact of this component [13]. Such peculiar mixed neoplasms can be composed of an adenoma with different degrees of dysplasia associated with either a NET of a different grade (from G1 to G3) or a NEC. The former are indolent neoplasms with no reported tumor-related deaths [60], while the latter are highly aggressive neoplasms with their behavior related to the NEC component [41]. Recent data suggest that mixed neoplasms composed of an adenoma associated with either a NET or a NEC are clonally related, supporting the speculative possibility to include these neoplasms into the MiNEN category [57].

### MiNENs of the Lung

#### Diagnostic Criteria and Classification

In the lung, the term “combined carcinoma” is used to define variants of pulmonary NECs and includes two different entities: (i) NECs composed of both small and large cell NECs and (ii) small or large cell NECs combined with a non-neuroendocrine component, irrespective of the amount of each component [15]. Unlike the digestive system, combined carcinomas of the lung do not include the rare cases in which the neuroendocrine component is represented by a NET (carcinoid), which although rare, has been reported in the literature [41]. It has also recently been demonstrated that in these peculiar mixed neoplasms, the two components are clonally related, suggesting that they should be considered true mixed neoplasms and not collision tumors [61]. In light of these recent findings and in order to be in line with the recent proposition of a common classification framework to unify the terminology of NENs arising in different organs [46], the term combined carcinoma may be critically revised, and a change of terminology seems appropriate. Consequently, the term MiNEN can be used to define mixed lung neoplasms composed of a neuroendocrine (either NEC or NET) and a non-neuroendocrine (adenocarcinoma or squamous cell carcinoma) component, as recently proposed [41].

#### Mimickers and Differential Diagnosis

As discussed above for MiNENs of the digestive system, there are some mimickers that need to be differentiated from true lung MiNENs. It is also worth noting that lung adenocarcinomas can show interspersed neuroendocrine cells when investigated with immunohistochemistry for general neuroendocrine markers, and they should not be considered MiNENs. Amphicrine carcinomas of the lung, although rare, have been reported, and they do not belong to the MiNEN category since they do not show different tumor components but are composed of cells showing a neuroendocrine and exocrine differentiation in the same cells [62]. From a clinical point of view, amphicrine carcinomas of the lung are well characterized and further studies are needed to better define this entity.
